# Contribution and Compensation Effects of Refracting Components to Ocular Aberrations in Keratoconus

**DOI:** 10.1167/iovs.66.11.64

**Published:** 2025-08-26

**Authors:** Satish K. Gupta, Andrew Carkeet, Scott A. Read, Stephen J. Vincent, David A. Atchison

**Affiliations:** 1 Centre for Vision and Eye Research, Queensland University of Technology, Brisbane, Queensland, Australia

**Keywords:** aberrometer, compensation, contact lenses, cornea, keratoconus, lens, Pentacam, raytracing, retinal image quality, Zernike aberrations

## Abstract

**Purpose:**

The purpose of this study was to determine the (i) contributions of refracting components to ocular aberrations and (ii) compensation effects exhibited by these components in keratoconus.

**Methods:**

Right eyes of 14 keratoconus and 20 control participants were analyzed using 5 mm pupils. Ocular aberrations were measured with a Hartmann-Shack aberrometer. Corneas were imaged with a Scheimpflug tomographer. Three-dimensional models of the total cornea and anterior cornea were created. Raytracing included correct object-image conjugates and corneal decentration relative to the aberrometer pupillary center to determine the total corneal and anterior corneal aberrations. Posterior corneal and lenticular aberrations were computed. Compensation effects (%) were calculated: 100 (anterior corneal−total corneal aberration)/anterior corneal aberration, and 100 (total corneal−ocular aberration)/total corneal aberration.

**Results:**

Considering coefficients for the total cornea with absolute values >0.05 µm, for both corneal surfaces, keratoconus had higher magnitudes than controls for C(2,−2), C(3,−3), C(3,−1), C(4,−2), total root mean square (RMS), higher-order RMS (HORMS), and *J*_45_. Both surfaces’ RMS aberrations were approximately 2 to 5 times higher in keratoconus than in controls. Anterior corneal RMS aberrations were approximately 5 times (keratoconus) and approximately 3 to 4 times (controls) higher than those of the posterior cornea. Posterior corneal compensations for anterior corneal aberrations were higher in keratoconus than in controls for C(3,−3) (21%, decompensation of −14%), C(3,−1) (21%, −33%), C(4,−2) (27%, −10%), C(4,+2) (22%, 10%), HORMS (20%, 2%), and *J*_0_ (68%, 66%), as were lenticular compensations for total corneal aberrations for C(2,−2) (40%, −64%), C(2,+2) (70%, 60%), total RMS (21%, 20%), and *J*_0_ (642%, −55%).

**Conclusions:**

Keratoconic eyes exhibited higher anterior and posterior corneal aberrations than control eyes. The posterior cornea and lens compensated partly for the anterior cornea and total cornea, respectively, with greater percentage compensations in keratoconus.

Keratoconus is a bilateral, usually asymmetric, noninflammatory corneal disorder, characterized by progressive thinning and ectasia resulting in conical protrusion of the cornea.^1^ These anatomical changes substantially increase ocular aberrations, particularly coma, which is considerably greater in eyes with manifest^2^^,^^3^ and forme fruste keratoconus^4^ than in non-keratoconic eyes. These aberrations are reduced with rigid contact lenses,^5^^–^^11^ which in conjunction with the post-lens tear layer mask the irregular anterior corneal surface. However, best-corrected visual acuity,^10^^,^^12^ contrast sensitivity,^12^^,^^13^ retinal image quality,^14^ and vision-related quality of life^15^^–^^18^ are still poorer in keratoconic eyes fitted with rigid lenses than in healthy eyes, which may be due to internal aberrations of the posterior cornea^19^^–^^21^ and crystalline lens.^21^^–^^24^

Early studies of component contributions to aberrations calculated the difference between the ocular and anterior corneal aberrations, but did not differentiate between the posterior cornea and the lens.^24^^–^^31^ Some studies that used the Oculus Pentacam Scheimpflug imaging system found higher posterior than anterior corneal aberrations in both keratoconus and controls (keratoconus > controls), with the aberrations having the same signs for both surfaces (Anand S, et al. IOVS 2008;49:ARVO E-Abstract 1031).^32^^,^^33^ These findings were incorrect, as the instrument's software used the same refractive index difference of 0.376 across both anterior and posterior corneal boundaries. Other studies reported lower posterior than anterior corneal aberrations.^19^^–^^21^^,^^34^^–^^39^ These studies used various mathematical techniques, such as Fourier decomposition of the corneal surfaces,^34^ raytracing through the surfaces,^21^^,^^35^^–^^39^ and comparing the surface shapes with ideal aberration-free shapes.^19^^,^^20^ Some of these studies considered the posterior cornea's object conjugate to be at infinity,^20^^,^^34^^,^^38^ rather than at the image formed by the anterior cornea. As the refraction at the anterior cornea affects the object conjugate for the posterior cornea, this approach may give inaccurate estimates of posterior corneal aberrations.^21^ Atchison et al.^21^ demonstrated in healthy eyes that using a distant object for the posterior cornea in ray tracing overestimates the posterior corneal aberrations (approximately −40% of anterior corneal aberrations), whereas using the correct object distance reveals they are, in fact, much smaller (–5% to −17% of anterior corneal aberrations). In addition, Kozhaya et al.^39^ calculated the difference between the ocular and total corneal aberrations and termed them non-corneal intraocular aberrations (lenticular aberrations). They found that intraocular higher-order aberrations were larger in keratoconic eyes than non-keratoconic myopic eyes. Their study lacked alignment to a common pupillary center reference for the two instruments used to determine the ocular aberrations (aberrometer) and corneal aberrations (anterior segment optical coherence tomographer), but rather they were referenced to the corneal vertex, which is useful if comparing the corneal aberrations between the instruments, but not for comparing ocular and corneal aberrations.

Another important factor to consider when determining aberrations is how corneal decentration is measured and defined. Some studies referenced the corneal data to the corneal sighting center^19^ or primary line of sight,^20^^,^^39^ whereas others have relied on the geometric center/axis,^37^^,^^38^ optical axis,^35^^,^^36^ or corneal vertex (Anand S, et al. IOVS 2008;49:ARVO E-Abstract 1031).^32^ Carkeet et al.^40^ highlighted the importance of accurately assessing pupil positions when correcting aberrations, as even small misalignments in pupillary reference can induce significant amounts of unintended aberrations, particularly in eyes with higher amount of aberrations such as in keratoconus.^41^ Atchison et al.^21^ demonstrated in healthy eyes that the incorrect use of corneal decentration affects oblique astigmatism, horizontal coma, and horizontal trefoil. It has been suggested that the corneal topographic center or corneal vertex, the origin of topography calculations, should be corrected to the pupillary center (as measured by an aberrometer or topographer)^21^^,^^30^^,^^42^ unless a combined corneal topographer and aberrometer instrument is used with a common pupillary reference center.^43^^–^^45^

Atchison et al.^21^ considered the decentration of corneal data relative to the aberrometer's pupillary center and the correct object conjugate for the posterior corneal surface. In young healthy eyes with a 5 mm pupil, the highest corneal aberration coefficients (absolute values >0.05 µm) were for horizontal astigmatism, horizontal coma, and spherical aberration. The anterior corneal root mean square (RMS) aberrations (total RMS_No defocus_ and higher-order RMS [HORMS]) were approximately three times higher than those of the posterior cornea. The posterior cornea compensated partially for the anterior corneal aberrations.

In summary, previous studies have considered ocular aberrations and the anterior corneal aberrations in keratoconus. Only a few studies have considered the posterior corneal and lenticular aberrations, but with either incorrect object/image conjugates or corneal decentration. Uncorrected aberrations from the posterior cornea and lens may explain the relatively poor visual performance obtained in many eyes with keratoconus fitted with the spherical rigid contact lenses. Therefore, it is important to determine the aberrations that arise from different ocular components.

This study investigated the contributions of the different refracting components to ocular aberrations in keratoconic and non-keratoconic eyes using raytracing in a modeling procedure. Correct object/image conjugates for the posterior corneal surface were used and corneal decentration relative to the aberrometer pupillary center was considered. The aberration compensation effects (%) exhibited by the ocular refracting components were also determined.

## Methodology

### Participants

The study was approved by the Queensland University of Technology (QUT) Human Research Ethics Committee and all procedures adhered to the tenets of the Declaration of Helsinki. Written informed consent was obtained from all participants. Pre-existing data from 40 participants aged between 20 and 40 years were retrieved, 20 with keratoconus and 20 age-matched controls. None had ocular pathology other than keratoconus with corneal scarring ≤ grade 1 (according to McMahon et al.’s grading scale).^46^ Six participants with keratoconus were excluded, as three had missing or incomplete corneal elevation data and three had pupil sizes <5 mm during aberrometry measurements.

Two keratoconus participants wore corneal rigid contact lenses full-time and seven of the control participants wore soft contact lenses. They discontinued lens wear for at least 1 day before measurements. Each participant underwent an ophthalmic examination to determine ocular health, subjective refraction, and axial length (IOLMaster 500 non-contact optical biometer; Carl Zeiss, Oberkochen, Germany). Measurements were performed without cycloplegia for the right eye of control participants and for both eyes of keratoconus participants. Only the right eye data of keratoconus participants were analyzed.

### Ocular Examinations

Anterior segment tomography was performed using the Pentacam, a rotating Scheimpflug camera (Oculus Optikgeräte GmbH, Wetzlar, Germany). Each scan captured 50 cross-sectional images to generate a 3-dimensional map of the anterior segment. Any map flagged as unreliable according to the instrument's quality specification was repeated until five reliable maps were obtained. Custom software was used to average the five Pentacam maps for each participant. The average axial radii of curvature for both anterior (*r*_1_) and posterior (*r*_2_) corneal surfaces, anterior chamber depth (ACD), central corneal thickness (CCT), and pupillary center offset relative to the corneal vertex (corneal topographic center) were determined from the averaged Pentacam data. The radii of curvature were extracted along 3 mm (0.1 mm step size) of each of 256 hemi-meridians for the anterior and posterior corneal surfaces. Each hemi-meridian was separated by 1.4 degrees. For each hemi-meridian, 30 radii of curvature were averaged, and the mean across all meridians was determined for each corneal surface. For each participant, the *r*_1_, *r*_2_, ACD, and CCT were used to calculate the longitudinal position of entrance pupil from the anterior cornea and the back vertex focal lengths for the total cornea and the anterior cornea (described later in the Methods section).

Ocular aberrations were measured with a Complete Ophthalmic Analysis System – High Definition (COAS-HD) Hartmann-Shack aberrometer (Wavefront Sciences Inc., Albuquerque, NM, USA). The instrument's multi-buffer acquisition mode was used to obtain Zernike polynomial aberration coefficients up to the sixth order. For each participant, a minimum of 6 series of measurements, each consisting of 10 frames, were obtained. An average of 74 frames were captured, of which approximately 90% were considered valid with pupil sizes ≥5 mm. The customized software was used to determine the aberration coefficients from averages of valid frames for 5 mm pupils using the method described by Schwiegerling.^47^ An experienced examiner obtained all measurements on the same day, within minutes, in the same room, and under the same mesopic room illumination (approximately 5 lux) to maintain a relatively large pupil size.

### Determination of Corneal and Lenticular Aberrations

For each participant, 5 elevation files, each containing 5 anterior corneal and 5 posterior corneal elevation maps in a square-grid format of 0.1 mm step size, were exported from the Pentacam. The data were averaged using customized software to produce the average anterior and posterior elevation maps, that were restricted to a 12.0 mm diameter. The Pentacam grid-sag data were manipulated using a custom program in MATLAB software, version R2023b (The MathWorks Inc., Natick, MA, USA) to the form required by the optical design software Zemax OpticStudio, version 2023 R2.01 (Ansys Inc., Canonsburg, PA, USA). This involved arranging the sag values into a single column and saving as a data file. This was imported into the Zemax software as a grid-sag file. Raytracing through this used linear interpolation between points.

The elevation files exported from each Pentacam map included locations of pupillary center (*PC*_H1_ and *PC*_V1_) and coordinates of 256 locations (*N*) across the pupillary margin (*PM*_H1_ and *PM*_V1_) relative to corneal topographic centers (*TC*_H1_ and *TC*_V1_). For each map, the average pupillary semi-diameter *R*_1_ was calculated as the average of linear distances between the pupillary center and each 256 locations across the pupillary margin. The “2*R*_1_” was the Pentacam pupillary size *D*_1_, which was given by:

(1) $$\begin{array}{c} D_{1}=2\;\times \;\frac{\sum \sqrt{{\left(PM_{H1}-\;PC_{H1}\right)}^{2}+\;{\left(PM_{V1}-\;PC_{V1}\right)}^{2}}}{N}\; \end{array}$$

The pupillary center locations and pupil sizes were averaged over the five Pentacam maps. The average COAS-HD aberrometer pupil size *D*_2_ was obtained from the average of the valid frames, as described previously.

Pupil sizes differ between the Pentacam and aberrometer (Table 1), a situation which can lead to corresponding pupillary centers being different.^48^ Due to the lower luminance at which it operates, the pupil center location for the COAS-HD aberrometer is typically infero-temporal relative to the Pentacam pupil center.^48^^–^^50^ Because the multi-frame procedure use for obtaining ocular aberrations in the current study did not include front-on eye images, it was impossible to remap pupil centers on an individual basis. Instead, estimates were made of the pupil center shift required using a previous study^48^ of young participants (18–25 years) that reported rates of aberrometer pupil center shift of (−)0.022 mm temporally and (−)0.013 mm inferiorly for each mm increase in pupil size.

**Table 1.** Means ± Standard Deviations (SDs) of Parameters for Keratoconus and Control Groups

Parameters	Keratoconus (*n* = 14)	Controls (*n* = 20)	*P* Value
M : F	9 : 5	10 : 10	0.41^*^
Age, y	29.57 ± 4.69	29.60 ± 5.44	0.99
Sphere, D	−1.21 ± 3.11	−0.98 ± 1.45	0.79
Cylinder, D	**−2.84 ± 1.61**	**−0.64 ± 0.87**	**<0.001**
Spherical equivalent refraction, D	−2.63 ± 3.05	−1.29 ± 1.55	0.15
Pentacam pupil size, mm	3.02 ± 0.63	3.05 ± 0.39	0.86
Aberrometer pupil size, mm	6.17 ± 0.89	6.48 ± 0.79	0.30
Horizontal corneal decentration, mm^†^	**0.21 ± 0.15**	**0.35 ± 0.16**	**0.02**
Vertical corneal decentration, mm^†^	**−0.20 ± 0.15**	**−0.02 ± 0.12**	**<0.001**
Central corneal thickness *d*_1_, mm	**0.48 ± 0.03**	**0.53 ± 0.03**	**<0.001**
Anterior chamber depth *d*_2_, mm	3.30 ± 0.32	3.09 ± 0.35	0.08
Axial length, mm	24.26 ± 0.98	24.16 ± 0.83	0.76
Anterior average radius of curvature *r*_1_, mm	**7.35 ± 0.37**	**7.85 ± 0.23**	**<0.001**
Posterior average radius of curvature *r*_2_, mm	**6.02 ± 0.34**	**6.53 ± 0.23**	**<0.001**
Entrance pupil position from anterior cornea *l*, mm	3.24 ± 0.33	3.06 ± 0.34	0.13
Back vertex focal length of anterior cornea, mm^‡^	**26.41 ± 1.34**	**28.19 ± 0.84**	**<0.001**
Back vertex focal length of total cornea, mm	**29.39 ± 1.48**	**31.30 ± 0.91**	**<0.001**

Statistically significant differences between keratoconus and control groups are in bold face.

* Chi-square test.

† Relative to aberrometer pupillary center.

‡ Relative to posterior cornea.

**Figure 1.**
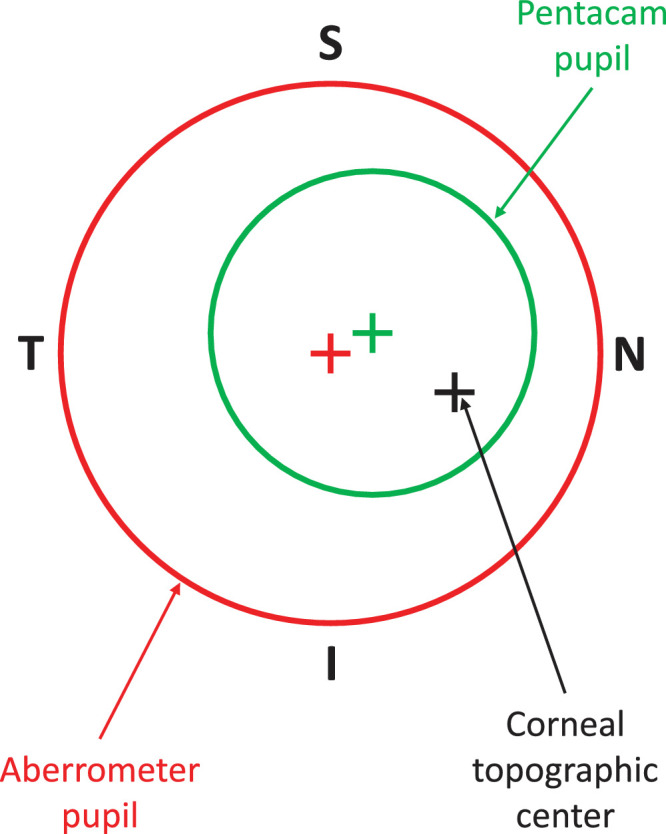
Corneal decentrations for the right eye. Effects are exaggerated for clarity. “S,” “I,” “N,” and “T” indicate superior (+y), inferior (–y), nasal (+x), and temporal (–x) directions, respectively. The corneal topographic center, the Pentacam pupil center, and the aberrometer pupil center are represented by the *black*, *green*, and *red crosses*, respectively. Here, the corneal topographic center is inferior (–ve) and nasal (+ve) to both the Pentacam and aberrometer pupillary centers.

Corneal decentration for the right eye is shown in Figure 1, where the Pentacam corneal topographic center is decentered in the inferior-nasal direction relative to both the aberrometer and Pentacam pupillary centers.

The eye's entrance pupil, which is the image of the aperture stop in the cornea, was set to be the stop of the system. Its longitudinal position from the anterior cornea *l* was determined by backward paraxial raytracing from the stop, assumed to be at the anterior surface of the lens. The equation for this is:

(2) $$\begin{array}{c} l={\left[\frac{L_{2}}{1+\frac{d_{1}L_{2}}{n_{1}}}\;-\;\frac{\left(n_{1}\;-\;n_{0}\right)}{r_{1}}\right]}^{-1} \end{array}$$

where the reduced object vergence *L*_2_ at the posterior cornea is given by:

(3) $$\begin{array}{c} L_{2}=n_{2}/d_{2}-\left(n_{2}-n_{1}\right)/r_{2} \end{array}$$

and *n*_0_, *n*_1_, and *n*_2_ are the refractive indices of air, the cornea, and aqueous humor, respectively, *d*_1_ and *d*_2_ are the thicknesses of the central cornea (CCT) and ACD, respectively, and *r*_1_ and *r*_2_ are the average radii of curvatures of the anterior and posterior corneal surfaces, respectively.

Using the *r*_1_ and *r*_2_, back vertex focal lengths of an anterior corneal surface model and a total corneal model were determined. Using Zemax OpticStudio, three-dimensional models of these two corneal systems were created. Each model was composed of an object and five surfaces. The object was at infinity, a flat stop of 5.0 mm diameter coincided with the entrance pupil position, a coordinate break incorporated the corneal topographic decentration relative to the aberrometer pupillary center, the anterior corneal surface was imported as a grid-sag data file at a distance of negative entrance pupil from the stop, and the posterior corneal surface was imported as a grid-sag data file at a distance *d*_1_ (CCT) from the anterior surface. For each model, the image surfaces or planes were the corresponding focal planes. For the anterior corneal surface and total cornea models, the refractive indices of the image space were 1.376 and 1.336, respectively. Raytracing was performed, and the Zernike aberration coefficients $C_{n}^{m}$ up to the sixth order were determined for 5 mm pupils in accordance with the International Ophthalmic Standard.^51^

Posterior corneal aberration coefficients $C{{}_{n}^{m}}_{P}$ were calculated by subtracting the coefficients of the anterior corneal surface model $C{{}_{n}^{m}}_{A}$ from the coefficients of the total cornea model $C{{}_{n}^{m}}_{T}$. Similarly, the lenticular aberration coefficients $C{{}_{n}^{m}}_{L}$ were calculated by subtracting the coefficients of the total corneal model $C{{}_{n}^{m}}_{T}$ from the ocular aberration coefficients $C{{}_{n}^{m}}_{O}$.

For each aberration coefficient, the compensation of the anterior cornea by the posterior cornea (*Comp*._*A*,*P*_) was the ratio of the difference between $C{{}_{n}^{m}}_{A}$ and $C{{}_{n}^{m}}_{T}$ to $C{{}_{n}^{m}}_{A}$. Similarly, the compensation of the total corneal aberrations by the lens (*Comp*._*T*,*L*_) was the ratio of the difference between $C{{}_{n}^{m}}_{T}$ and $C{{}_{n}^{m}}_{O}$ to $C{{}_{n}^{m}}_{T}$:

(4) $$\begin{array}{c} Comp._{A,P}\;=\;\frac{\bar{C{{}_{n}^{m}}_{A}}\;-\;\bar{C{{}_{n}^{m}}_{T}}}{\bar{C{{}_{n}^{m}}_{A}}}\;\times \;100\% \end{array}$$

(5) $$\begin{array}{c} Comp._{T,L}\;=\;\frac{\bar{C{{}_{n}^{m}}_{T}}\;-\;\bar{C{{}_{n}^{m}}_{O}}}{\bar{C{{}_{n}^{m}}_{T}}}\;\times \;100\% \end{array}$$

where the bars above the symbols indicate that the means of coefficients in a group (keratoconus or control) are being used. Positive values indicate compensation and negative values indicate decompensation. These equations were used also to quantify compensation for RMS of aberrations. This approach to determine the compensation effects was based on previous studies.^21^^,^^25^

### Statistical Analysis

The SPSS version 29.0 (IBM Corp., Armonk, NY, USA), was used. The mean Zernike aberration coefficients, for all ocular components, were reported for the second to fourth orders since coefficients for the fifth to sixth orders, except for $C_{5}^{-1}$, were not clinically significant (<0.05 µm)^21^ for ocular, total cornea, and anterior cornea. We found that the average of standard deviations of the mean ocular aberration coefficients for the second to sixth order (excluding defocus) in the control group was 0.06 µm, which supports a 0.05 µm cutoff as being reasonable to report clinically significant aberrations. Zernike piston $C_{0}^{0}$ and tilts ($C_{1}^{-1}$ and $C_{1}^{+1}$) were excluded because they do not affect image quality.^52^ The Zernike defocus $C_{2}^{0}$ and defocus-dependent parameters were also excluded because in the raytracing modeling procedure, the image planes were the corneal focal planes, whereas the aberrometer uses the retina as ocular image plane. The total RMS of aberrations (excluding defocus) and Zernike refractions (*J*_0_ and *J*_45_) were analyzed for the second to sixth orders. The HORMS of aberrations was analyzed for the third to sixth orders.

The Shapiro–Wilk test indicated that most ocular parameters and 15 aberration parameters (11 aberration coefficients for the second to fourth orders except defocus, total RMS, HORMS, *J*_0_, and *J*_45_) for all ocular components were not distributed differently from normal distributions (*P* > 0.05) in both the keratoconus and control groups, and thus parametric tests were used. Independent samples *t*-tests determined if the differences in ocular parameters and aberration parameters between the keratoconus and control groups were statistically significant. Linear regression and Pearson's correlation coefficient *r* were used to determine the relationships and correlations, respectively, between the ocular components for each aberration parameter. As multiple comparisons were made for 15 aberration parameters, a Bonferroni correction was applied to reduce the risk of false positive results, so that *P* values < 0.0033 (from 0.05/15) were considered statistically significant.

To determine the repeatability of the instruments used in this study, the metrics used were: (i) Pentacam: mean of 5 anterior corneal and 5 posterior corneal surface sag measurements (5 mm diameter), and (ii) aberrometer: mean of all valid frames’ ocular total RMS aberrations for 5 mm pupil size (up to eighth order excluding piston, tilt, and defocus). In addition, for each metric in the keratoconus and control groups, the mean within-subject standard deviation (S_w_) of repeated measurements was calculated as: √ (∑ σ^2^/n), where “*σ*” is the mean of SDs for “*n*” repeated measurements, and the average proportion of measurement variation from the mean value was calculated as: (S_w_/mean) × 100%.

## Results

The mean ± standard deviation (SD) age of both the keratoconus (*n* = 14, 9 males) and the control groups (*n* = 20, 10 males) were 30 ± 5 years. The spherical equivalent refractions (SERs) were −2.6 ± 3.1 diopter (D; sphere = +2.75 D to −9.00 D and cylinder = −0.75 D to −5.75 D) for the keratoconus group and −1.3 ± 1.6 D (+0.50 D to −4.25 D and 0.00 D to −3.00 D) for the control group (*P* = 0.15). Keratoconic eyes had more corneal astigmatism than control eyes (–2.8 ± 1.6 D vs. −0.6 ± 0.9 D, *P* < 0.001), thinner corneas (0.48 ± 0.03 mm vs. 0.53 ± 0.03 mm, *P* < 0.001), and smaller average radii of curvature for both anterior (7.35 ± 0.37 mm vs. 7.85 ± 0.23 mm, *P* < 0.001) and posterior (6.02 ± 0.34 mm vs. 6.53 ± 0.23 mm, *P* < 0.001) corneal surfaces. Values for other ocular parameters are given in Table 1.

### Corneal Decentration

The mean ± SD decentration of the corneal topographic center relative to the aberrometer's pupillary center was estimated to be +0.21 ± 0.15 mm nasally and −0.20 ± 0.15 mm inferiorly in the keratoconus group, and +0.35 ± 0.16 mm nasally and −0.02 ± 0.12 mm inferiorly in the control group. The estimated vertical corneal decentration was greater in the keratoconus group (*P* < 0.001), whereas horizontal corneal decentration was greater in the control group (*P* = 0.02).

### Aberrations: Keratoconus Group Versus Control Group

**Figure 2.**
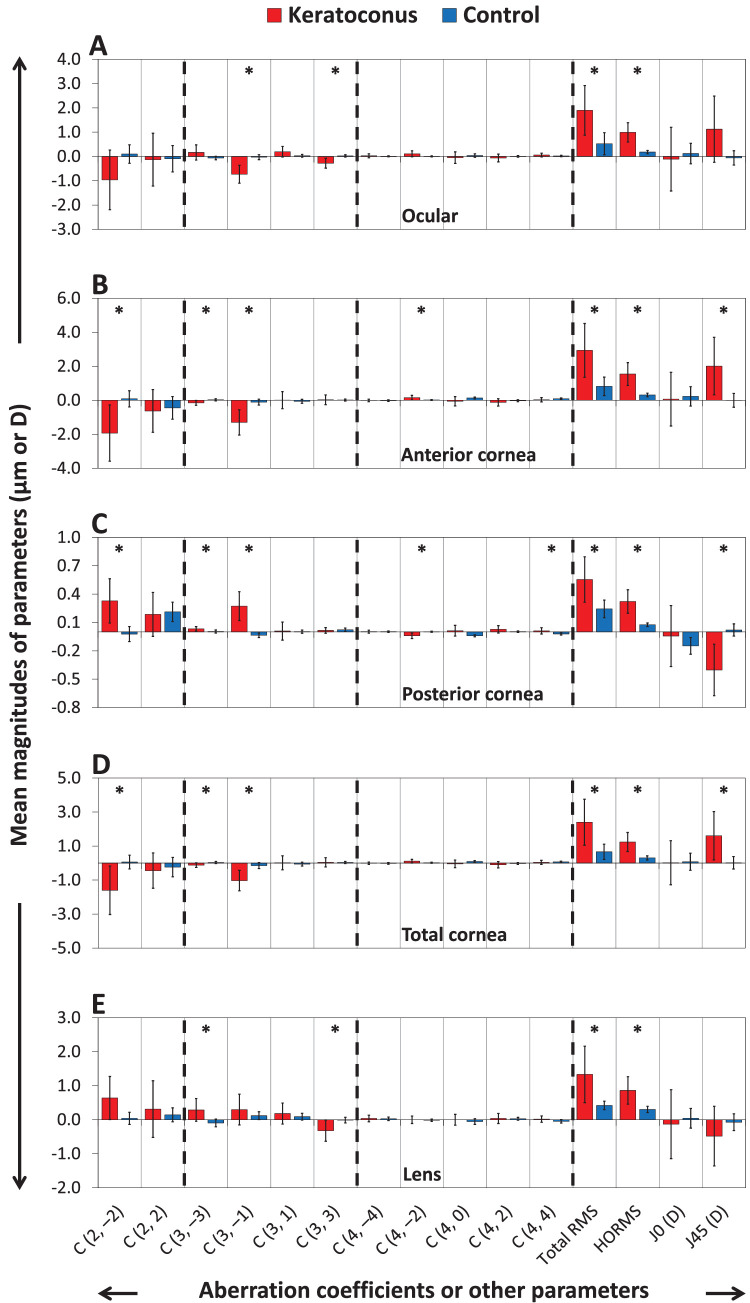
Means ± SDs of aberration parameters up to the fourth order for ocular components. (**A**) Ocular, (**B**) anterior cornea, (**C**) posterior cornea, (**D**) total cornea, and (**E**) lens in the keratoconus (*red bars*) and control (*blue bars*) groups. The vertical scales for each panel include the minimum and maximum values (including error bars). The *dashed black lines* separate aberration parameters by order. *Statistically significant differences between the keratoconus and control groups (*P* < 0.05/15).

Table 2 and Figure 2 show the mean ± SD of the Zernike aberration parameters up to the fourth order for the ocular aberrations and their contributions in the keratoconus and control groups. For all components, the keratoconus group had greater absolute magnitudes of aberrations than the control group. Ocular aberrations were significantly higher in the keratoconus group than in the control group for primary vertical coma $C_{3}^{-1}$, primary horizontal trefoil $C_{3}^{+3}$, total RMS, and HORMS (all *P* ≤ 0.001; see Fig. 2A). The ocular total RMS and HORMS aberrations were higher in the keratoconus group than in the control group by approximately 4 times and 5 times, respectively.

**Table 2.** Means ± SDs of Aberration Parameters for Ocular Refracting Components in Keratoconus and Control Groups

			Ocular		Anterior Cornea		Posterior Cornea		Total Cornea		Lens	
S.N.	Aberration Parameters	Unit, µm or D	Keratoconus	Control	Keratoconus	Control	Keratoconus	Control	Keratoconus	Control	Keratoconus	Control
1	$C_{2}^{-2}$	µm	−0.96 ± 1.23	0.10 ± 0.38	**−1.93 ± 1.65**	**0.08 ± 0.48**	**0.33 ± 0.24**	**−0.02 ± 0.08**	**−1.60 ± 1.42**	**0.06 ± 0.40**	0.64 ± 0.63	0.04 ± 0.18
2	$C_{2}^{+2}$	µm	−0.13 ± 1.09	−0.09 ± 0.54	−0.63 ± 1.26	−0.45 ± 0.66	0.19 ± 0.23	0.21 ± 0.10	−0.44 ± 1.04	−0.24 ± 0.57	0.31 ± 0.83	0.14 ± 0.21
3	$C_{3}^{-3}$	µm	0.17 ± 0.31	−0.06 ± 0.07	**−0.15 ± 0.14**	**0.03 ± 0.07**	**0.03 ± 0.02**	**0.004 ± 0.02**	**−0.12 ± 0.14**	**0.03 ± 0.06**	**0.29 ± 0.33**	**−0.10 ± 0.11**
4	$C_{3}^{-1}$	µm	**−0.73 ± 0.37**	**−0.03 ± 0.10**	**−1.30 ± 0.74**	**−0.11 ± 0.17**	**0.27 ± 0.15**	**−0.04 ± 0.02**	**−1.03 ± 0.60**	**−0.15 ± 0.18**	0.30 ± 0.45	0.12 ± 0.12
5	$C_{3}^{+1}$	µm	0.20 ± 0.22	0.03 ± 0.06	0.01 ± 0.50	−0.06 ± 0.12	0.01 ± 0.10	0.002 ± 0.02	0.02 ± 0.41	−0.06 ± 0.11	0.18 ± 0.31	0.09 ± 0.10
6	$C_{3}^{+3}$	µm	**−0.28 ± 0.20**	**0.02 ± 0.05**	0.03 ± 0.29	0.01 ± 0.06	0.02 ± 0.03	0.02 ± 0.02	0.04 ± 0.27	0.04 ± 0.06	**−0.32 ± 0.31**	**−0.01 ± 0.08**
7	$C_{4}^{-4}$	µm	0.03 ± 0.08	0.01 ± 0.02	−0.01 ± 0.08	−0.02 ± 0.05	0.002 ± 0.02	0.002 ± 0.01	−0.01 ± 0.07	−0.02 ± 0.05	0.04 ± 0.10	0.03 ± 0.05
8	$C_{4}^{-2}$	µm	0.11 ± 0.12	0.003 ± 0.02	**0.15 ± 0.13**	**0.02 ± 0.03**	**−0.04 ± 0.03**	**0.002 ± 0.01**	0.11 ± 0.11	0.02 ± 0.03	−0.002 ± 0.11	−0.01 ± 0.03
9	$C_{4}^{0}$	µm	−0.05 ± 0.24	0.04 ± 0.07	−0.06 ± 0.27	0.13 ± 0.05	0.01 ± 0.06	−0.04 ± 0.01	−0.05 ± 0.22	0.09 ± 0.05	−0.001 ± 0.16	−0.05 ± 0.08
10	$C_{4}^{+2}$	µm	−0.06 ± 0.16	0.004 ± 0.03	−0.13 ± 0.21	−0.03 ± 0.06	0.03 ± 0.04	0.003 ± 0.01	−0.10 ± 0.19	−0.02 ± 0.06	0.04 ± 0.15	0.03 ± 0.04
11	$C_{4}^{+4}$	µm	0.06 ± 0.08	0.02 ± 0.03	0.03 ± 0.12	0.09 ± 0.04	**0.01 ± 0.03**	**−0.02 ± 0.01**	0.04 ± 0.12	0.07 ± 0.05	0.02 ± 0.09	−0.05 ± 0.05
12	Total RMS^*^	µm	**1.90 ± 1.02**	**0.53 ± 0.45**	**2.93 ± 1.58**	**0.82 ± 0.54**	**0.55 ± 0.24**	**0.24 ± 0.09**	**2.40 ± 1.35**	**0.66 ± 0.45**	**1.33 ± 0.83**	**0.42 ± 0.13**
13	HORMS^*^	µm	**0.99 ± 0.40**	**0.18 ± 0.07**	**1.54 ± 0.67**	**0.31 ± 0.11**	**0.32 ± 0.13**	**0.08 ± 0.02**	**1.24 ± 0.56**	**0.30 ± 0.12**	**0.86 ± 0.40**	**0.30 ± 0.09**
14	*J*_0_ ^*^	D	−0.11 ± 1.31	0.12 ± 0.42	0.06 ± 1.58	0.23 ± 0.57	−0.04 ± 0.32	−0.15 ± 0.09	0.02 ± 1.30	0.08 ± 0.51	−0.13 ± 1.01	0.04 ± 0.29
15	*J*_45_ ^*^	D	1.12 ± 1.36	−0.06 ± 0.29	**2.01 ± 1.69**	**−0.01 ± 0.41**	**−0.40 ± 0.27**	**0.02 ± 0.07**	**1.61 ± 1.42**	**0.02 ± 0.37**	−0.48 ± 0.88	−0.07 ± 0.25

* Calculated for the second to sixth orders.

Statistically significant differences between keratoconus and control groups are in bold face (*P* < 0.05/15).

S.N., serial number.

For the anterior cornea, the parameters that were significantly higher in the keratoconus group than in the control group were primary oblique astigmatism $C_{2}^{-2}$, primary oblique trefoil $C_{3}^{-3}$, primary vertical coma $C_{3}^{-1}$, secondary oblique astigmatism $C_{4}^{-2}$, total RMS, HORMS, and *J*_45_ (all *P* ≤ 0.002; see Fig. 2B). Similar findings were observed for the posterior corneal parameters for $C_{2}^{-2}$, $C_{3}^{-3}$, $C_{3}^{-1}$, $C_{4}^{-2}$, quatrefoil $C_{4}^{+4}$, total RMS, HORMS, and *J*_45_ (all *P* ≤ 0.003; see Fig. 2C).

The anterior corneal total RMS and HORMS were higher in the keratoconus group than in the control group by approximately 4 times and 5 times, respectively. The posterior corneal RMS was higher in the keratoconus group than in the control group by approximately 2 times for total RMS and by approximately 4 times for HORMS. In the keratoconus group, both anterior corneal total RMS and HORMS were approximately five times higher than corresponding posterior corneal RMS, whereas in the control group, these proportions were approximately three times for total RMS and approximately four times for HORMS.

For the total corneal parameters, the keratoconus group had higher magnitudes than the control group for $C_{2}^{-2}$, $C_{3}^{-3}$, $C_{3}^{-1}$, total RMS, HORMS, and *J*_45_ (all *P* ≤ 0.001; see Fig. 2D). The RMS aberrations were higher in the keratoconus group than in the control group by approximately four times for both total RMS and HORMS.

The lenticular aberrations that were statistically higher in the keratoconus group than in the control group were $C_{3}^{-3}$, $C_{3}^{+3}$, total RMS, and HORMS (all *P* ≤ 0.003; see Fig. 2E).

### Corneal and Lenticular Contributions to Ocular Aberrations

The signs of the mean coefficients and refraction terms for the ocular and total corneal aberrations were the same for 9 of 13 parameters in the keratoconus group and 8 of 13 parameters in the control group (see Table 2). Table 3 shows the correlations between ocular refracting components in the groups.

**Table 3.** Pearson's Correlation Coefficients (r) Between Ocular Refracting Components in the Keratoconus and Control Groups

			Ocular vs. Total Cornea		Ocular vs. Lens		Total Cornea vs. Lens		Anterior vs. Posterior Cornea	
S.N.	Aberration Parameters	Unit, µm or D	Keratoconus	Control	Keratoconus	Control	Keratoconus	Control	Keratoconus	Control
1	$C_{2}^{-2}$	µm	**0.90 (<0.001)**	**0.90 (<0.001)**	−0.08 (0.79)	0.09 (0.69)	−0.51 (0.06)	−0.35 (0.13)	**−0.99 (<0.001)**	**−0.91 (<0.001)**
2	$C_{2}^{+2}$	µm	0.69 (0.01)	**0.93 (<0.001)**	0.44 (0.11)	0.05 (0.84)	−0.34 (0.23)	−0.31 (0.18)	**−0.96 (<0.001)**	**−0.93 (<0.001)**
3	$C_{3}^{-3}$	µm	0.05 (0.87)	−0.43 (0.06)	**0.92 (<0.001)**	**0.86 (<0.001)**	−0.36 (0.21)	**−0.83 (<0.001)**	−0.39 (0.17)	−0.19 (0.41)
4	$C_{3}^{-1}$	µm	0.66 (0.01)	**0.79 (<0.001)**	−0.07 (0.81)	−0.33 (0.16)	**−0.79 (0.001)**	**−0.84 (<0.001)**	**−0.93 (<0.001)**	0.08 (0.73)
5	$C_{3}^{+1}$	µm	0.68 (0.01)	0.48 (0.03)	−0.21 (0.47)	0.02 (0.94)	**−0.86 (<0.001)**	**−0.87 (<0.001)**	**−0.94 (<0.001)**	−0.16 (0.50)
6	$C_{3}^{+3}$	µm	0.15 (0.61)	−0.10 (0.67)	0.51 (0.06)	**0.66 (0.002)**	**−0.77 (0.001)**	**−0.82 (<0.001)**	−0.50 (0.07)	0.06 (0.81)
7	$C_{4}^{-4}$	µm	0.19 (0.52)	0.19 (0.42)	0.72 (0.004)	0.18 (0.44)	−0.55 (0.04)	**−0.93 (<0.001)**	−0.52 (0.06)	0.28 (0.24)
8	$C_{4}^{-2}$	µm	0.56 (0.04)	0.16 (0.51)	0.58 (0.03)	0.51 (0.02)	−0.35 (0.22)	**−0.77 (<0.001)**	**−0.94 (<0.001)**	0.50 (0.03)
9	$C_{4}^{0}$	µm	**0.77 (0.001)**	0.14 (0.55)	0.43 (0.12)	**0.77 (<0.001)**	−0.25 (0.39)	−0.52 (0.02)	**−0.94 (<0.001)**	0.03 (0.90)
10	$C_{4}^{+2}$	µm	0.65 (0.01)	**0.66 (0.002)**	0.26 (0.37)	−0.06 (0.81)	−0.57 (0.04)	**−0.79 (<0.001)**	**−0.74 (0.003)**	−0.27 (0.25)
11	$C_{4}^{+4}$	µm	0.60 (0.02)	0.10 (0.68)	0.08 (0.80)	0.50 (0.03)	**−0.76 (0.002)**	**−0.82 (<0.001)**	−0.35 (0.22)	−0.12 (0.63)
12	Total RMS^*^	µm	**0.94 (<0.001)**	**0.89 (<0.001)**	0.60 (0.02)	0.28 (0.24)	0.60 (0.02)	0.37 (0.11)	**0.96 (<0.001)**	**0.84 (<0.001)**
13	HORMS^*^	µm	**0.76 (0.002)**	0.46 (0.04)	0.64 (0.01)	0.46 (0.04)	**0.84 (<0.001)**	**0.77 (<0.001)**	**0.94 (<0.001)**	0.38 (0.10)
14	*J*_0_ ^*^	D	0.70 (0.01)	**0.82 (<0.001)**	0.40 (0.16)	0.03 (0.90)	−0.38 (0.19)	−0.55 (0.01)	**−0.90 (<0.001)**	**−0.77 (<0.001)**
15	*J*_45_ ^*^	D	**0.80 (0.001)**	**0.74 (<0.001)**	0.25 (0.38)	0.09 (0.69)	−0.37 (0.19)	−0.60 (0.01)	**−0.99 (<0.001)**	**−0.69 (0.001)**

* Calculated for the second to sixth orders.

Statistically significant correlations are in bold face (*P* < 0.05/15).

**Figure 3.**
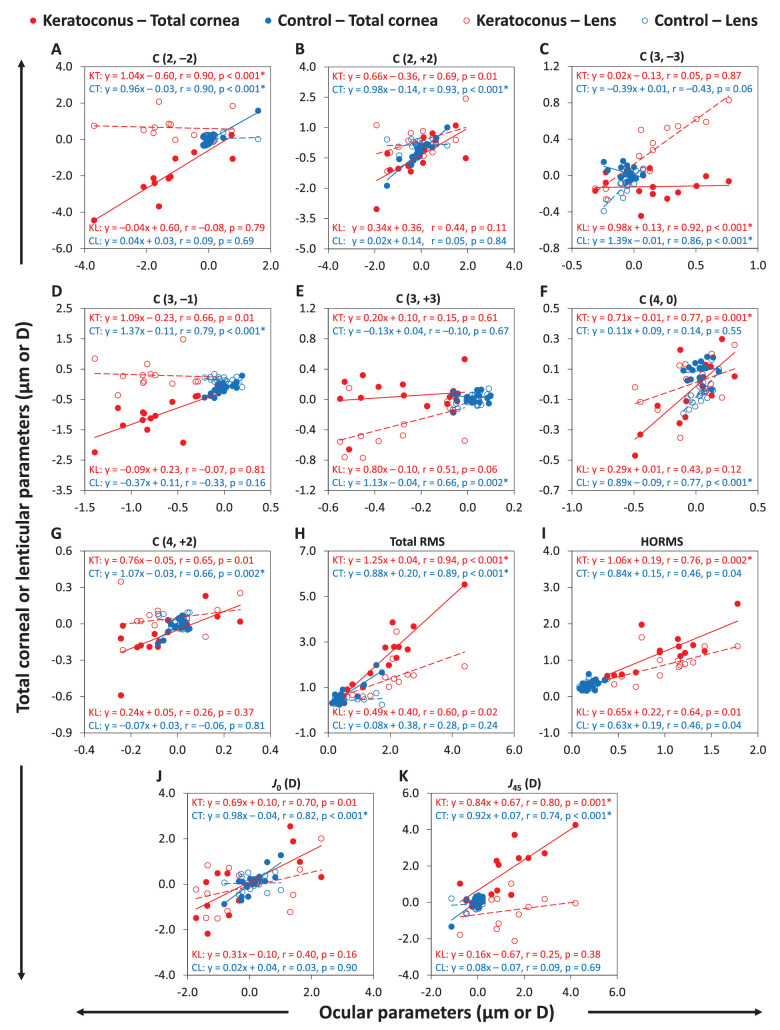
Linear regressions of correlations of the ocular parameters with corresponding total corneal and lenticular parameters. *Red* and *blue filled circles* are data points (total cornea) for the keratoconus (KT) and control (CT) groups, respectively. *Red* and *blue unfilled circles* are data points (lens) for the keratoconus (KL) and control (CL) groups, respectively. Regression lines (*solid lines* for total cornea and *dashed lines* for lens) and equations with statistics are included. (**A**) Primary oblique astigmatism, (**B**) primary with-/against-the-rule astigmatism, (**C**) primary oblique trefoil, (**D**) primary vertical coma, (**E**) primary horizontal trefoil, (**F**) primary spherical aberration, (**G**) secondary with-/against-the-rule astigmatism, (**H**) total RMS, (**I**) HORMS , (**J**) *J*_0_, and (**K**) *J*_45_. *Statistically significant correlations (*P* < 0.05/15).

Figure 3 shows the linear regressions for the ocular with the total cornea and lens for parameters that were statistically significant in either the keratoconus or control groups. Significant positive correlations were found between the ocular and total corneal parameters (5/15 parameters in keratoconus and 7/15 parameters in controls; see Table 3).

Similarly, the signs of the mean aberration coefficients and refraction terms for the ocular and lenticular aberrations were the same for 7 of 13 parameters in the keratoconus group and for 7 of 13 parameters in the control group (see Table 2). Significant positive correlations were found between the ocular and lenticular parameters (1/15 parameters in keratoconus and 3/15 parameters in controls; see Table 3).

### Compensation for Aberrations

**Figure 4.**
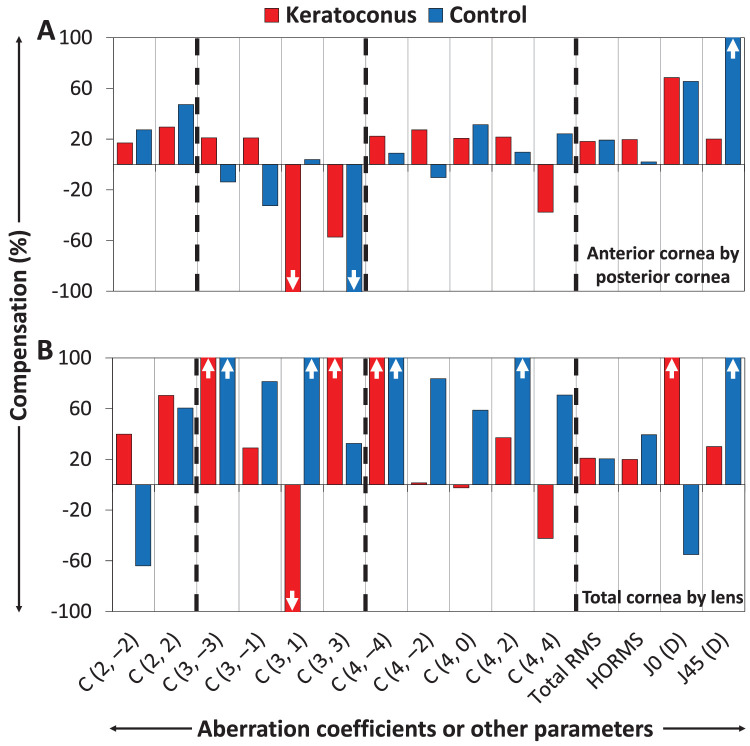
(**A**) Compensation (%) of the anterior corneal parameters by the posterior cornea, and (**B**) compensation of the total corneal parameters by the lens in the keratoconus (*red bars*) and control (*blue bars*) groups. The positive and negative proportions across both panels indicate compensation and decompensation, respectively. “↑” and “↓” in a few bars, respectively, indicate compensation or decompensation >100%. The *dashed black lines* separate aberration parameters by order.

The means of the anterior corneal and posterior corneal aberration coefficients and refraction terms had the opposite signs for 10 of 13 parameters in the keratoconus group and 9 of 13 parameters in the control group (see Table 2). The posterior cornea compensated for the anterior corneal parameters in the keratoconus (12/15 parameters) and control (11/15 parameters) groups. Of the parameters for which the total cornea had mean absolute values >0.05 µm (clinically significant) in either of the study group, the percentages of posterior corneal compensation were higher in the keratoconus group than in the control group for $C_{3}^{-3}$ (21% vs. decompensation of −14%), $C_{3}^{-1}$ (21% vs. −33%), $C_{4}^{-2}$ (27% vs. −10%), $C_{4}^{+2}$ (22% vs. 10%), HORMS (20% vs. 2%), and *J*_0_ (68% vs. 66%; see Fig. 4A). For all 15 aberration parameters, the proportions of compensation and decompensation effects of the anterior cornea by the posterior cornea and of the total cornea by the lens in the keratoconus and control groups are provided in the supplementary information (Supplementary Table S1).

**Figure 5.**
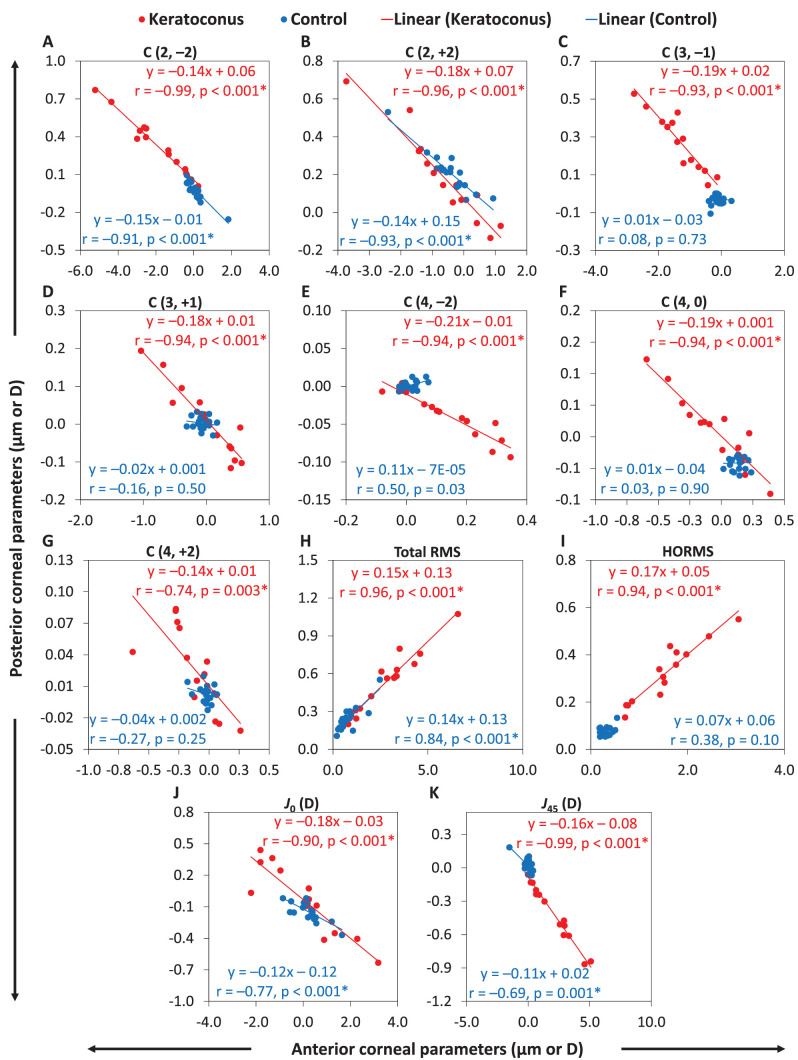
Linear regressions of correlations of the anterior corneal parameters with corresponding posterior corneal parameters. *Red* and *blue filled circles* are data points for the keratoconus and control groups, respectively. Regression lines and equations with statistics are included. (**A**) Primary oblique astigmatism, (**B**) primary with-/against-the-rule astigmatism, (**C**) primary vertical coma, (**D**) primary horizontal coma, (**E**) secondary oblique astigmatism, (**F**) primary spherical aberration, (**G**) secondary with-/against-the-rule astigmatism, (**H**) total RMS, (**I**) HORMS, (**J**) *J*_0_, and (**K**) *J*_45_. *Statistically significant correlations (*P* < 0.05/15).

Table 3 shows several significant negative correlations between the anterior and posterior corneal aberration coefficients and refraction terms (9/13 parameters in the keratoconus group and 4/13 parameters in the control group). Figure 5 shows linear regressions of correlations between the anterior corneal and posterior corneal surfaces for the parameters that were statistically significant in either the keratoconus or control groups.

The means of the total corneal and lenticular aberration coefficients and refraction terms had the opposite signs for 10 of 13 parameters in the keratoconus group and for 11 of 13 parameters in the control group (see Table 2). The lens compensated for the total corneal aberrations in the keratoconus (12/15 parameters) and control (13/15 parameters) groups. Of the clinically significant total corneal aberration coefficients, the percentages of lenticular compensation were higher in the keratoconus group than in the control group for $C_{2}^{-2}$ (40% vs. −64%), $C_{2}^{+2}$ (70% vs. 60%), total RMS aberrations (21% vs. 20%), and *J*_0_ (642% vs. −55%; see Fig. 4B). Table 3 shows significant negative correlations between the total corneal and lenticular aberration coefficients and refraction terms (4/13 parameters in the keratoconus group and 8/13 parameters in the control group).

### Repeatability of the Pentacam and the Aberrometer

The mean anterior corneal sags for the 5 Pentacam measurements were 0.305 mm and 0.284 mm in the keratoconus and control groups, respectively. The corresponding means S_w_ were similar, at 0.195 mm and 0.182 mm. Similarly, the mean posterior corneal sags in the keratoconus and control groups, respectively, were 0.383 mm with a mean S_w_ of 0.245 mm and 0.347 mm with a mean S_w_ of 0.226 mm. For the anterior cornea, the average proportions of measurement variation from the mean values were approximately 64% in both the keratoconus and control groups. For the posterior cornea, these proportions were approximately 64% in the keratoconus group and approximately 65% in the control group. This shows that the mean S_w_ and proportional repeatability of the Pentacam sag measurements have similar values for the keratoconic and control eyes, and for both corneal surfaces.

For the aberrometer, the mean ocular total RMS aberrations were 1.907 µm (mean S_w_ = 0.049 µm) and 0.532 µm (mean S_w_ = 0.015 µm), respectively, in the keratoconus and control groups. The keratoconic eyes exhibited approximately three times higher mean S_w_ than the control eyes, although the average proportions of repeatability from the mean values were similar (approximately 3%) in both the keratoconic and control eyes.

## Discussion

This study investigated the magnitudes of aberrations in keratoconus, the contributions of different refracting components to the aberrations, and the compensation effects between refracting components. To obtain accurate estimates of aberrations, factors such as the correct object/image conjugates for corneal surfaces and corneal topography decentration relative to the aberrometer pupil center were considered. Aberrations up to the sixth order were determined in keratoconic and non-keratoconic eyes for a 5 mm pupil size without cycloplegia.

Given that pre-existing data were used in the current study, prospective sample size calculation was not possible. Therefore, we performed a post hoc power calculation using the G*Power software (version 3.1.9.7) for the 14 keratoconic eyes and 20 controls in our study. The mean ± SD ocular total RMS of 1.90 ± 1.02 µm in the keratoconus group and 0.53 ± 0.45 µm in the control group (see Table 2) gave an effect size of 1.74, which with an alpha error probability of 0.05 gave an estimated study power of 99%.

Excluding defocus and defocus-dependent parameters, several aberrations were significantly different between the keratoconus and control groups for all refracting components. The total RMS and HORMS aberrations for each refracting component were higher in the keratoconus group than in the control group by approximately two to five times. Kozhaya et al.^39^ found that the HORMS for each refracting component were higher in the keratoconus group than in the control group by approximately three to nine times. Nakagawa et al.^20^ found that the anterior corneal and posterior corneal HORMS were higher in the keratoconus group than in the control group by approximately seven to nine times. Chen and Yoon^19^ reported higher magnitudes of the anterior corneal and posterior corneal HORMS in the advanced keratoconus than in the normal eyes by approximately five to eight times, but the differences reduced to three to four times for moderate keratoconus and disappeared for mild keratoconus.

The RMS aberrations for the anterior cornea were greater than those for the posterior cornea by approximately five times in the keratoconus group, similar to the results of Kozhaya et al.,^39^ Nakagawa et al.,^20^ and Chen and Yoon^19^ for HORMS. In the control group, these proportions were approximately three to four times, similar to those found by previous studies for HORMS^19^^,^^20^ and RMS aberrations,^21^ but lower than the six times reported by Kozhaya et al.^39^ for HORMS.

Of the clinically significant total corneal aberration coefficients, the percentage posterior corneal compensations for the anterior corneal aberrations were higher in the keratoconus group than in the control group for the higher-order aberrations (HOAs) such as oblique trefoil (21% vs. −14%), vertical coma (21% vs. −33%), oblique astigmatism (27% vs. −10%), secondary with-/against-the-rule astigmatism (22% vs. 10%), HORMS aberrations (20% vs. 2%), and *J*_0_ (68% vs. 66%). Decompensation of >100% was observed for horizontal coma in the keratoconus group and for horizontal trefoil in the control group (dominated by 4 control participants exhibiting high decompensation of >100%; see Fig. 4A). In addition, individual participant analysis showed compensation of horizontal coma and horizontal trefoil, respectively, in all 14 keratoconus participants and in 8 of 20 control participants. In healthy eyes, Atchison et al.^21^ found compensations of 39%, 28%, and 26% (incorrectly given as 43%) for horizontal astigmatism, horizontal coma, and spherical aberration, respectively.

**Figure 6.**
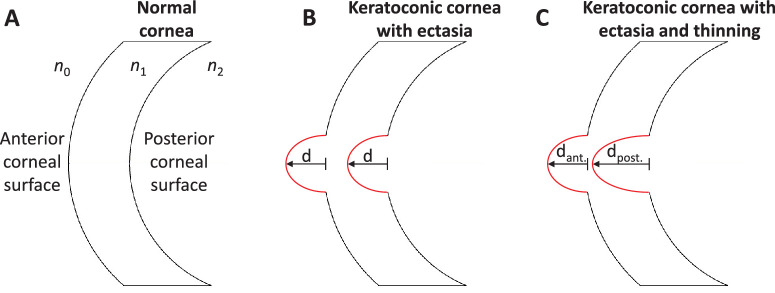
Explanation of why higher proportional compensation of the anterior corneal aberrations by the posterior cornea is expected in keratoconus. (**A**) Normal cornea, (**B**) keratoconic cornea with the same amount of ectasia “d” at both the anterior corneal and posterior corneal surface, where approximately 11% of the compensation effect is observed, and (**C**) keratoconic corneal thinning, where the posterior corneal ectasia d_post._ is more than the anterior corneal ectasia d_ant._ resulting in a higher percentage of compensation effect in a keratoconic eye than its counterparts.

For keratoconus, the greater proportional HOA compensation, of the anterior cornea by the posterior cornea, arises due to considerable structural changes such as steeper posterior surface curvature (see Table 1). Figure 6 explains why higher proportional compensation of the anterior corneal aberrations by the posterior cornea is observed in keratoconus. The posterior cornea deforms alongside the anterior cornea because they are interconnected through stromal collagen fibers.^19^^,^^53^^,^^54^ The same amount of keratoconic ectasia *d* at both the anterior corneal and posterior corneal surfaces make the cornea weak and distorted, but localized corneal thinning does not occur (see Fig. 6B). Therefore, the compensation of a wavefront passing into the anterior cornea *W*_*ant*. _ by a wavefront leaving the posterior cornea *W*_*post*. _ through the point of ectasia is given by:

(6) $$\begin{array}{c} W_{post.\;}=W_{ant.}\times \frac{\left(n_{2}-n_{1}\right)}{\left(n_{1}-n_{0}\right)} \end{array}$$

where *n*_0_, *n*_1_, and *n*_2_ are the refractive indices of air, the cornea, and aqueous humor, respectively. Solving Equation 6 gives approximately 11% compensation in keratoconic ectasia. Further, in keratoconus with corneal thinning, the posterior corneal ectasia *d_post._* is greater than the anterior corneal ectasia *d_ant._* (see Fig. 6C), resulting in a higher proportion of compensation.

Additionally, we remodeled the total corneal and anterior corneal systems to determine what effect referencing the corneal topography to the topographer pupil center, rather than the aberrometer pupil center, would have on our results. The mean ± SD corneal decentration relative to the Pentacam pupillary center was +0.14 ± 0.15 mm nasally and −0.25 ± 0.15 mm inferiorly in the keratoconus group, and +0.27 ± 0.16 mm nasally and −0.07 ± 0.11 mm inferiorly in the control group. For the anterior cornea in the keratoconus group, changing the pupillary reference from the aberrometer to the Pentacam caused a change in the mean total RMS and the mean HORMS of +1.7% and −1%, respectively. Corresponding changes in the control group were −0.4% and −2.1%. The mean posterior corneal aberrations in the keratoconus group changed by +1.4% (total RMS) and −1.0% (HORMS) and by +1.6% (total RMS) and +1.2% (HORMS) in the control group. This indicates that the small pupillary center shifts between the two instruments had mild effects on both the anterior corneal and posterior corneal RMS aberrations in both the keratoconus and control groups.

Of the clinically significant total corneal aberration coefficients, the percentage lenticular compensations for the total corneal aberrations were also higher in the keratoconus group than in the control group, but only for the lower-order aberrations (LOAs) such as oblique astigmatism and with-/against-the-rule astigmatism, and total RMS aberrations and *J*_0_. Decompensation of >100% was observed for horizontal coma in the keratoconus group (see Fig. 4B), which was dominated by 3 keratoconus participants exhibiting high decompensation of >100%. In addition, individual participant analysis showed compensation for horizontal coma in 10 of 14 keratoconus participants.

The finding of higher lenticular aberrations in the keratoconus group than in the control group was unexpected but is consistent with the study by Kozhaya et al.^39^ One possible reason is that it is an artifact, with either one or both Pentacam tomographer^55^ and Hartmann–Shack wavefront aberrometer^56^ not being able to measure highly aberrated eyes accurately. In our study, the mean S_w_ values of repeated Pentacam measurements of the anterior corneal and posterior corneal sags were similar between the keratoconus and control groups, whereas the mean S_w_ values of repeated aberrometer measurements of the ocular total RMS aberrations were approximately three times higher in the keratoconic eyes than in the control eyes. Given that some aberration coefficients have very low corneal values in comparison with the ocular values, such as $C_{3}^{+1}$ and $C_{3}^{+3}$ (see Table 2), it would be more likely to be the latter. There may be a passive mechanism, with an increase in the angle lambda (λ) between the line of sight and the pupillary axis, such that increased corneal aberrations are compensated by increasing lenticular aberrations, as found by Artal et al.^25^ for healthy corneas. From the corneal decentration divided by the entrance pupil depth (the entrance pupil being the stop in our modeling), the vertical component of angle lambda in our study was +0.5 ± 2.2 degrees for the control group and +3.7 ± 3.0 degrees for the keratoconus group, thus indicating considerable scope for a passive mechanism to operate in the keratoconic eyes for aberrations like vertical coma $C_{3}^{-1}$.

It has also been reported that the line of sight shifts relative to the pupillary center in keratoconus^57^ may induce a shift in ocular alignment giving rise to coma aberrations.^58^ Consequently, the internal optics of the eye (majorly lenticular) was found to compensate for the corneal aberrations, particularly in eyes with large angle kappa (κ) between the visual axis and the pupillary axis and with large pupillary decentration.^25^^,^^59^^,^^60^ All of these lenticular effects appear to be passive in nature and depend on the severity of the disease.

We have used the terms “corneal” and “lenticular” to describe the aberrations. Artal and colleagues^23^^–^^27^ used the terms “corneal” and “internal” where the former term referred to the anterior cornea and the latter term referred to the combined effects of the posterior cornea and lens. Kozhaya et al.^39^ used the terms “corneal” and “non-corneal intraocular” (or simply “non-corneal”). One argument for the latter approach is that the lenticular aberrations are not being determined directly, but as differences between ocular and corneal aberrations. In addition, the cornea will influence the aberrations at the lens through the reduced vergence, tilts, and decentrations the former provides.

The involvement of the lens in producing aberrations and addressing whether lenticular aberrations are accurately derived by subtracting total corneal aberrations from ocular aberrations, could be investigated by assessing a large sample of eyes with well centered intraocular lenses (IOLs) with minimal tilt. If the IOL parameters are known, it should be possible to accurately estimate the contribution of lenticular aberrations. Subtracting the total corneal HOAs from the measured ocular HOAs would indicate whether the determination of spherical aberration and other HOAs are accurate. Related studies for investigating the accuracy of determined lenticular aberration contributions include assessment of patients with post-hyperopic LASIK (laser in situ keratomileusis) treatments in which the posterior cornea and lens can be assumed to be unchanged, as well as patients with lenticonus and presumably normal anterior and posterior corneal shapes.

Although the approach to determine compensation given by Equations 4 and 5 is not ideal because it does not provide any measure of individual variance and so it is difficult to estimate a confidence limit, it was considered preferable to averaging the participant compensation in a group. For the latter approach, small aberration magnitudes can distort the compensation results. For example, if an individual had $C{{}_{n}^{m}}_{A}$ = −0.0001 µm and $C{{}_{n}^{m}}_{T}$ = 0.001 µm, Equation 4 gives a compensation of 1100% of the anterior corneal aberration by the posterior cornea.

In keratoconus, rigid contact lenses with spherical front surfaces minimize anterior corneal aberrations by reducing the refractive index difference of 0.376 between the cornea and air to 0.04 between the cornea and the post-lens tear film but leave the posterior corneal aberrations uncorrected. This may explain the clinical observation that keratoconic eyes corrected with front surface spherical rigid contact lenses still exhibit suboptimal visual performance.^10^^,^^12^^–^^14^ In such cases, optimizing the front surface asphericity may be beneficial, however, decentration of an aspheric contact lens on a keratoconic eye can be detrimental to visual performance.^61^^–^^63^

This study has some limitations:

(i) As mentioned earlier in the Discussion section, the instruments, particularly the aberrometer, may not be able to measure highly aberrated eyes accurately.
(ii) There was no direct determination of lenticular aberrations, but these were determined as differences between the ocular and the total corneal aberrations.
(iii) As emphasized earlier, the individual pupillary center shifts between corneal tomographer and aberrometer were not available, and were estimated using the average data from a previous study.^48^ For most participants, we found that small pupillary shifts have minimal impact on the anterior and posterior corneal RMS aberrations, but there might be considerable pupillary shifts and larger aberration changes in some participants.^49^
(iv) We used different instruments to determine corneal tomography and ocular aberrations. These would have different principles of alignment that might affect the results. The use of a single instrument to do both operations, such as the newer Oculus Pentacam AXL Wave, would improve matters and would enable the measurements to be taken much closer together, although the different requirements of the two processes might still be a confounding factor.
(v) The tomographer was a Scheimpflug device and it is likely that newer anterior segment optical coherence tomographers provide better axial resolution (for instance, <10 µm with the Anterion swept-source optical coherence tomography [OCT]^55^).
(vi) There was a difference in location at which the anterior corneal and total corneal aberrations were determined. If one was dealing with the Seidel aberration theory,^64^ which is valid for rotationally symmetric systems and small amounts of aberrations, the contribution of each surface could be determined by analyzing the deviation of the optical path length from that for a perfect optical system during Gaussian raytracing. The best thing that can be done in the present situation with highly aberrated eyes with non-rotationally symmetric surfaces is to add one component (the posterior cornea) to another (the anterior location) to see how the aberrations change at similar reference positions. Here, these reference positions are the exit pupils. For the control group in our study, these were 3.66 ± 0.35 mm and 3.62 ± 0.35 mm relative to the anterior corneal surface for the anterior corneal model and total corneal model, respectively. For the keratoconus group, the corresponding positions were 3.82 ± 0.32 mm and 3.77 ± 0.32 mm. Thus, the exit pupil positions were close at 0.04 to 0.05 mm apart for the 2 models.
(vii) Like previous studies, fixed refractive indices were used, that is, 1.376 for the cornea and 1.336 for the aqueous humor. There is probably a slight axial decrease in indices from corneal front to back,^65^ but not enough is known about this to include it in modeling at present.

## Conclusions

Keratoconic eyes had higher magnitudes of aberration parameters than controls for all ocular refracting components. Overall, the RMS aberrations for each component were higher in the keratoconus group than in the control group by approximately two to five times. The posterior cornea and lens compensated partially for the anterior corneal HOAs and total corneal LOAs, respectively, with larger percentage compensation effects in the keratoconus group than in the control group. The contributions and compensation effects of different refracting components of the eye to ocular aberrations should be considered when assessing and correcting aberrations in keratoconus.

## Supplementary Material

Supplement 1
