# Supplementary material for: Contribution and Compensation Effects of Refracting Components to Ocular Aberrations in Keratoconus
Source: Invest Ophthalmol Vis Sci. 2025 Aug 26;66(11):64. doi: 10.1167/iovs.66.11.64 (PMC12393118; doi:10.1167/iovs.66.11.64)
Supplement: Supplement 1 [file iovs-66-11-64_s001.pdf]

Supplementary information

Supplementary Table S1: Overall compensation effects (%) of the anterior corneal parameters by the posterior cornea, and of the total corneal parameters by the lens in the keratoconus and control groups.

**S.N.**	**Aberration parameters**	**Compensation (%) of anterior corneal parameters by posterior cornea**		**Compensation (%) of total corneal parameters by lens**	
		**Keratoconus**	**Control**	**Keratoconus**	**Control**
1	C_2^{-2}	17	27	**40**	**–64**
2	C_2^{+2}	29	47	**70**	**60**
3	C_3^{-3}	**21**	**–14**	237	293
4	C_3^{-1}	**21**	**–33**	29	81
5	C_3^{+1}	–134	4	–1201	147
6	C_3^{+3}	–57	–182	761	33
7	C_4^{-4}	22	9	440	140
8	C_4^{-2}	**27**	**–10**	1	84
9	C_4^{0}	21	31	–2	59
10	C_4^{+2}	**22**	**10**	37	116
11	C_4^{+4}	–38	24	–42	71
12	Total RMS#	18	19	**21**	**20**
13	HORMS#	**20**	**2**	20	39
14	J_0#	**68**	**66**	**642**	**–55**
15	J_{45}#	20	402	30	479

#Calculated for the 2nd- to 6th-orders

Of the parameters for which the total cornea had mean absolute values > 0.05 µm (clinically significant) in either of the study group, the percentages (%) of compensation that were higher in the keratoconus group than in the control group are bolded.

The positive (+ve) and negative (–ve) percentages indicate compensation and decompensation, respectively.
